# Cytotoxic Tirucallane Triterpenoids from *Melia azedarach* Fruits

**DOI:** 10.3390/molecules15095866

**Published:** 2010-08-27

**Authors:** Nikoletta G. Ntalli, Filippo Cottiglia, Carlos A. Bueno, Laura E. Alché, Marco Leonti, Simona Vargiu, Ersilia Bifulco, Urania Menkissoglu-Spiroudi, Pierluigi Caboni

**Affiliations:** 1 Pesticide Science Laboratory, Faculty of Agriculture, Aristotle University of Thessaloniki, 54124 Thessaloniki, Greece; 2 Dipartimento Farmaco Chimico Tecnologico, University of Cagliari, via Ospedale 72, 09124 Cagliari, Italy; 3 Bueno, C.A. and Alché, L.E. Laboratorio de Virología, Departamento de Química Biológica, Facultad de Ciencias Exactas y Naturales, Universidad de Buenos Aires, Pabellón 2, 4to. Piso, Ciudad Universitaria, Buenos Aires, Argentina; 4 Department of Toxicology, Università degli Studi di Cagliari, via Ospedale 72, 09124 Cagliari, Italy

**Keywords:** methyl kulonate, 3-*α*-tigloylmelianol, melianone, 21-*β*-acetoxy-melianone, cytotoxicity

## Abstract

The phytochemical investigation of the dichloromethane-soluble part of the methanol extract obtained from the fruits of *Melia azedarach* afforded one new tirucallane-type triterpene, 3-*α*-tigloylmelianol (**1**) and three known tirucallanes, melianone (**2), **21-*β*-acetoxy-melianone (**3**), and methyl kulonate (**4**). The structure of the isolated compounds was mainly determined by 1D and 2D NMR experiments as well as HPLC-Q-TOF mass spectrometry. The cytotoxicity of the isolated compounds toward the human lung adenocarcinoma epithelial cell line A549 was determined, while no activity was observed against the phytonematode *Meloidogyne incognita.*

## 1. Introduction

*Melia azedarach* L. is a botanical species belonging to the family Meliaceae also known as Ku-lian, China tree or Chinaberry tree. It is native to Asia but is now found in parts of Northern Australia, Africa, North America, tropical South America and Southern Europe. In South America is commonly known as “paraiso” or paradise, and in the US as Indian lilac or white cedar. In Traditional Chinese Medicine, it is used as an antiparasitic and antifungal agent, but many of its constituent compounds have been found to exhibit a wide range of other biological properties [1,2,3,4,5,6,7,8,9,10,11,12]. The main group of biologically active compounds concentrated in Chinaberry’s fruits are called limonoids, consisting of a large group of lipophilic substances [13]. 

Limonoids are metabolically altered triterpenes and have a prototypical structure either containing or derived from a precursor with a 4,4,8-trimethyl-17-furanylsteroid skeleton. Of the 300 limonoids known today, about one third, also known as meliacins, are obtained from Meliaceae species (*Azadirachta indica* and *Melia azedarach*). Limonoids have attracted much attention due to their wide range of properties permitting both pharmacological and plant protection applications [1,2,10,13,14,15]. Some of the most important biological properties of the limonoids are the antioxidant [12], anticancer [11,16], antimalarial [17], anthelmintic [18], antibacterial [19], antifungal [6,7], and insecticidal effects against disease vectors [4] as well as insect pests [1,2,14]. From an ecological standpoint limonoids are not harmful to ecosystems because they are not toxic to parasitoids and predators [20,21] and they have a relatively short residual life [22,23]. Nevertheless, at high concentration levels they can be poisonous to humans [24]. In our previous study we have reported the nematicidal activity of the methanol (MeOH) extract obtained from the ripe fruits of *M. azedarach* against *Meloidogyne incognita* second stage juveniles (J2) [8]. In the present investigation we report on the chemical characterisation of the tirucallane triterpenoids isolated from the dichloromethane (DCM) soluble fraction of the MeOH extract, and on their individual activity against nematodes. We also report on the antiproliferative activity and effect of the purified compounds on viability of a lung adenocarcinoma epithelial cell line. 

## 2. Results and Discussion

### 2.1. Chemistry

**Figure 1:**
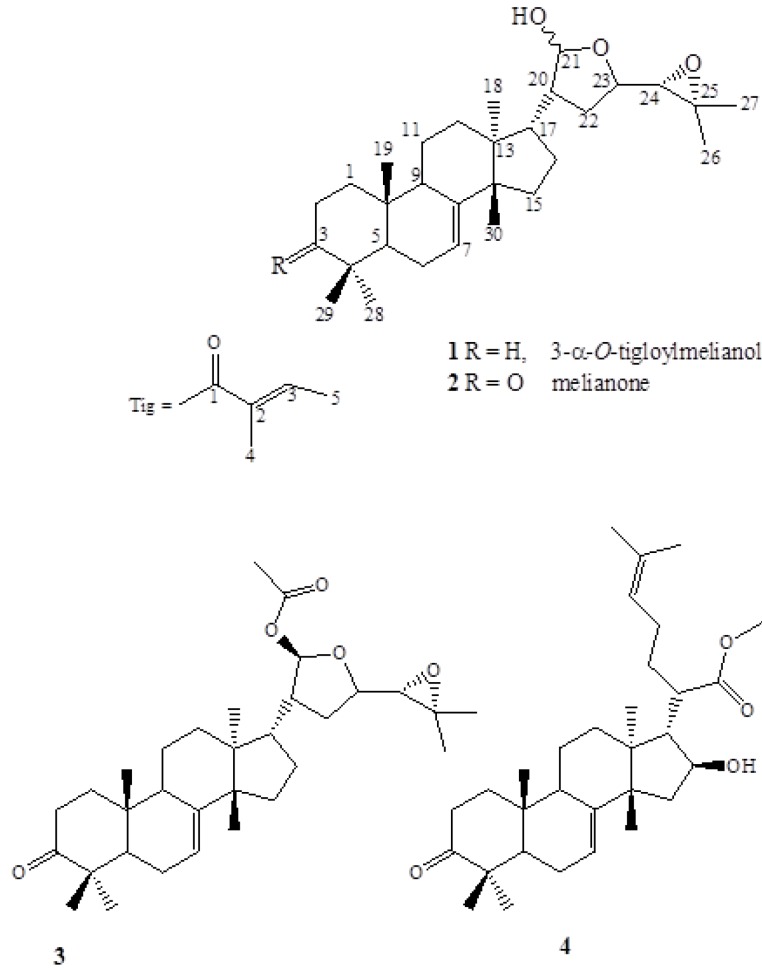
Structures of tirucallane triterpenoids from *M. azedarach.* 3-*α*-tigloylmelianol (**1**) and melianone (**2**), two known protolimonoids were also isolated and identified as 21-*β*-acetoxymelianone (**3**) and methyl kulonate (**4**).

Regarding the biological activities of triterpenoids the investigations need to focus on the optimization of extraction methods, detailed structural characterization, quantification, and structure activity delineation so that they can be safely introduced in our arsenal of pharmaceuticals used against diseases [13]. The phytochemical analysis of the DCM soluble fraction of the MeOH extract resulted in the identification of one new tirucallane triterpenoid **1** and three known ones **2**-**4** (Figure 1). The respective *M. azedarach* yields in triterpenoids expressed as dry fruits’ weight are presented in Table 1. All the isolated compounds were purified by means of open column chromatography.

**Table 1:** Yield of tirucallane triterpenoids from dried fruits of *Melia azedarach*.

Compound	Yield mg/kg^a ^(w/w)
Melianone	130
21-*β*-Acetoxymelianone	16
Methyl kulonate	35
3-*α*-Tigloylmelianol	78

^a^ Expressed as dry fruit weight.

**Figure 2:**
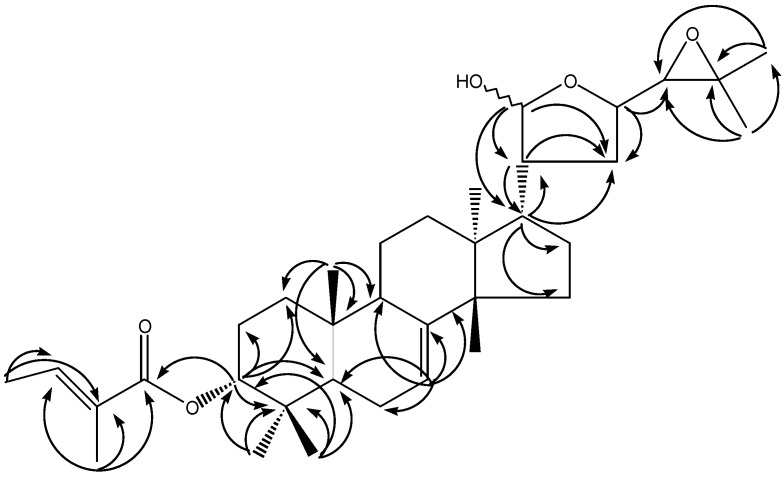
Key HMBC correlations (H→C) of compound **1**.

Compound **1** was obtained as an amorphous white powder. Its ESI MS displayed a pseudomolecular ion at *m/z* 577 [M + Na]^+^, whereas the HR-TOF-ESI MS revealed a pseudomolecular ion at *m/z* 555.4093 [M + H]^+^ (calc. 555.4044). The data were consistent with the molecular formula C_35_H_54_O_5._ The ^1^H-NMR spectrum showed ten tertiary (δ_H_ 0.76, 0.81, 0.90, 0.95, 0.96, 0.98, 1.27, 1.28, 1.29, 1.81) and one secondary methyl groups [δ_H_ 1.75 (d, *J* = 6.8 Hz)], two olefinic protons [δ_H_ 6.80 (qq, *J* = 6.8, 1.6 Hz), 5.24, m] and seven oxy-methines at δ 5.34 (d, *J* = 2.8 Hz), 5.29 (d, *J* = 2.8 Hz), 4.68 (br s), 4.10 (m), 4.15(m), 2.82 (d, *J* = 7.6 Hz), 2.67 (d, *J* = 7.6 Hz) (Table 2). Detailed analyses of ^1^H- and^ 13^C-NMR spectra revealed that **1** shared high structural similarity to melianone (**2**) [25] with the main difference between the two compounds being the presence of an oxy-methine at δ_H_ 4.68 (δ_C_ 78.3) at C-3 instead of the ketone group in melianone (**2**) (Table 2). The ^13^C-NMR spectrum of **1** (Table 2) exhibited five further signals at δ167.5, 136.6, 129.2, 14.4 and 12.2, which were sorted by DEPT 135 experiment into two CH_3_ (δ 14.4, 12.2 ppm), one CH (δ 136.6 ppm) and two quaternary carbons (δ 167.5, 129.2). The above mentioned carbons could be easily ascribable to a tiglate [26]. In the HMBC spectrum of **1 **(Figure 2) the correlation of δ_H_ 0.81 (H-28) and δ_H_ 0.95 (H-29) with δ_C_ 36.9 (C-4), 46.1 (C-5), 78.3 (C-3) and of δ_H_ 4.68 (H-3) with δ_C_ 167.5 (C-1′), 36.9 (C-4), 46.1 (C-5), 32.1 (C-1), 27.5 (C-2), 27.6 (C-28) and 21.4 (C-29) fixed the tigloyloxy group at the C-3 position of **1**. The broad singlet of H-3 suggested an α-orientation of the tigloyloxy moiety. This assignment was supported by correlation of δ_H _4.68 (H-3) with δ_H_ 076 (H-19) in the ROESY spectrum. The HSQC spectra of compounds **1** and **2** showed correlation of the C-21 group resonances at δ 97.8 and 101.8 with H-21 [at δ 5.29 (d, *J* = 2.8 Hz) and 5.33 (d, *J* = 2.8 Hz) for **1** and at δ 5.27 (d, *J* = 2.7 Hz) and 5.32 (d, *J* = 2.7 Hz) for **2**], characterising them as C-21 epimers. The structure of compound **1** was finally confirmed by extensive 2D NMR measurements as 3-*α*-tigloylmelianol (**1**).

Besides 3-*α*-tigloylmelianol (**1**) and melianone (**2**), two known protolimonoids were also isolated and identified as 21-*β*-acetoxymelianone (**3**) and methyl kulonate (**4**) (Figure 1) [26,27,28,29].

**Table 2:** ^1^H- and ^13^C-NMR data for compounds 1-2 (CDCl_3_, *δ* in ppm and *J* in Hz).

Position	1			2	
	δ_H_	δ_C_		δ_H_	δ_C_
1a	1.39 (m)	32.1			38.5
1b	167 (m)				
2	1.49 (m)	27.5		2.24 ddd (14.3, 14, 6.4)	35.1
	1.85 (m)			2.75 ddd (14.3, 3.6, 3.4)	
3	4.68 (s br)	78.3			216.8; 216.7
4		36.9			47.9
5	1.79 (m)	46.1		1.82 (m)	52.5; 52.4
6	1.73 (m)	22.9		1.70 (m)	23.3
7	5.23 (d, 2.4)	118.2; 118.1		5.28 br	118.2; 118.1
8		146.0; 145.9			145.8; 145.6
9	2.30 (t, 7.6); 2.2 (t, 7.6)	49.7; 48.8			49.6; 48.4
10		34.9			34.9
11	1.54 (m)	17.4			17.8
12a	1.53 (m)	35.3			35.2
12b	1.98 (m)				
13		43.8; 43.6			43.8; 43.6
4		50.9; 50.4			50.8; 50.5
15	1.55 (m)	34.2			34.3
16	1.61 (m)	27.5; 27.1			27.5; 27.3
17	2.01 (m); 2.04 (m)	47.1; 45.2		2.03 (m); 2.06 (m)	47.1; 45.2
18	0.90 (s)	23.9		0.80 (s); 085 (s)	24.6
19	0.76 (s)	13.0			12.8
20	1.71 (m)	33.8; 31.7			33.8; 31.7
21	5.33 (d, 2.8); 5.29 (d,2.8)	101.8; 97.8			101.8; 97.8
22	1.98 (m)	31.5; 31.3			31.5; 31.3
23	3.89 (m); 3.83 (m)	78.5; 77.0		3.88 (m); 3.84 (m)	78.5; 77.1
24	2.82 (d, 7.6); 2.67(d,7.6)	67.8; 65.3		2.81 (d, 7.6); 2.67 (d, 7.6)	67.8; 65.4
25		58.0; 57.2			58.0; 57.2
26^a)^	1.29 (s); 1.28 (s)	25.0; 24.9		1.29(s); 1.28 (s)	25.0; 24.9
27^a)^	1.27 (s)	19.4; 19.2		1.26 (s)	19.5; 19.2
28	0.81 (s)	27.6		0.97 (s)	24.4
29	0.95 (s)	21.4		0.98 (s)	21.6
30	0.98 (s); 0.96 (s)	22.6; 22.3		1.00 (s); 1.07 (s)	22.6
1′		167.5			
2′		129.2			
3′	6.80 (qq, 6.8, 1.6)	136.6			
4′	1.75 (d, 6.8)	14.4			
5′	1.81 (s)	12.2			

^a)^ values within any column may be interchanged.

### 2.2. Cytotoxic and antiproliferative activities

**Figure 3:**
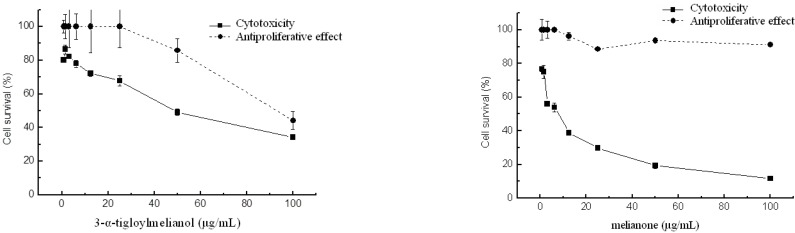
Regression curves of cytotoxic and antiproliferative activity of 3-*α*-tigloylmelianol and melianone against the tumorigenic cell line A549.

The literature concerning the cytotoxic properties of limonoids is extensive [10,13,16,30,31,32,33] and recent efforts are designed to investigate the cellular and molecular mechanisms by which such effects are exerted in the tumorigenic cell lines [34]. Nevertheless, the biological activity of the limonoids isolated and identified in this study is limited. Melianone possesses cytotoxic properties [29], acts against *Trypanosoma cruzi* (Protista) [35] and activates hepatic metabolic enzymes in rat [36]. Methyl kulonate is reported to have anticancer properties [33]. Herein, the human lung adenocarcinoma epithelial cell line A549 was chosen to determine the cytotoxic and antiproliferative activities of 21-*β*-acetoxymelianone, methyl kulonate, 3-*α*-tigloylmelianol and melianone. Human lung adenocarcinoma A549 cells were grown in MEM 10%. Cell cultures were maintained in a 4% CO_2_ humidified atmosphere at 37 ºC. When the cytotoxic activity was assayed, the percentage of surviving cells decreased gradually with the increase in the concentration of 21-*β*-acetoxymelianone, 3-*α*-tigloyl-melianol, and melianone after 24-hour incubation, whereas methyl kulonate showed no cytotoxicity at any of the concentrations tested (data not shown). The CC_50_ values of 21-*β*-acetoxymelianone, 3-*α*-tigloylmelianol and melianone were 90.6, 64.7 and 3.6 μg/mL, respectively, indicating that melianone exhibited the highest cytotoxic effect. Only 21-*β*-acetoxymelianone and 3-*α*-tigloylmelianol showed moderate antiproliferative effect, with IC_50_ values of 100 and 91.8 μg/mL. These results demonstrate that 21-*β*-acetoxymelianone and 3-*α*-tigloylmelianol gather both, cytotoxic and antiproliferative activities, although 3-*α*-tigloylmelianol proved to be more cytotoxic than 21-*β*-acetoxymelianone. Finally, neither cytotoxic nor antiproliferative activity was found for methyl kulonate (data not shown) and 3-*α*-tigloylmelianol exhibited no cytotoxic effect in a normal Vero cell line, derived from kidney epithelial cells of the African Green Monkey (Barquero A., personal communication). The antiproliferative and cytotoxic activity of 3-*α*-tigloylmelianol and melianone are presented in Figure 3.

### 2.3. Nematicidal activity

The isolated triterpenoids as well as limonin were evaluated for their nematicidal activity against *M. incognita*. All compounds had no significant effect on J2 motility after 4d of immersion in solutions at the dose range of 31.2–500 μg/mL (data not shown). These results are in accordance with our previous findings, where the limonoid azadirachtin was not found to possess nematicidal activity against *M. incognita* [37], and agree with the conclusion that *M. azedarach* nematicidal activity is not related to its limonoids contents.

## 3. Experimental

### 3.1. General

UV spectra were recorded on a GBC Cintra 5 spectrophotometer. Melting points were determined on a Köfler apparatus and are uncorrected. NMR spectra were recorded at 25 ºC on a Varian UNITY INOVA 400 MHz, operating at 400 MHz for ^1^H and 100 MHz for ^13^C, respectively. Compounds were measured in CDCl_3_ and the spectra referenced against residual non-deuterated solvents. LC-MS DAD analysis: low resolution MS was performed on a Varian tandem mass spectrometer (Paolo Alto, CA) consisting of a ProStar 410 autosampler, two ProStar 210 pumps, and a 1200 L triple quadrupole mass spectrometer. The column used was a Phenomenex GEMINI 3 μm C_18_ 110 A (100 × 4.6 mm). The mobile phase consisted of (A) 0.1% formic acid in water and (B) methanol. The solvent gradient started at 20% A and 80% B, reaching 100% B at 10 min, and it was held in these conditions up to 30 min. For the following 5 min the mobile phase was re-stabilized at 80% B and it remained at these conditions for 5 min before every successive injection. The mobile phase was pumped constantly at 0.4 mL/min and the injection volume was 20 μL. The substances revelation was achieved at a spectrum scan from 200 to 600 nm. The software used for the detector’s analysis parameters set and for the HPLC data analysis was ChromQuest Version 4.0 Build 1120 and HP Chem, Version Α.07.01 respectively. LC-MS analysis was conducted in electrospray ionization interface and the conditions, operated in the positive ion mode, were as follows: capillary potential, 55 V; shield potential, 775 V; needle voltage, 5700 V; nitrogen pressure, 54mTorr; housing temperature, 56 ºC; drying gas temperature, 380 ºC; scan time 1; detector multiplier voltage, 1200 V; isolation width of m/z 1.6 for the quadrupole 1; scan 100-1500 amu. HPLC-MS-Q-TOF Analysis: The isolated compounds were analyzed by reverse-phase HPLC on an Agilent 1200 series HPLC system fitted with a microchip technology using an Agilent Zorbax 300 SB-C18 5 μm, 43 mm × 75 μm (Agilent, Santa Clara, CA). The HPLC conditions were as follows: flow rate: 0.4 μL/min; solvent A: 0.1% formic acid in water; solvent B: acetonitrile; and gradient: solvent B 5–100% over 10 min. The samples (1 μL) were analyzed by ESI in positive mode using an Agilent 6520 Time of Flight (TOF) MS. Mass spectral data was acquired in the range, *m/z* 100–1500 with an acquisition rate of 1.35 spectra/s, averaging 10,000 transients. The source parameters were adjusted as follows: drying gas temperature 250 ºC, drying gas flow rate 5 L/min, nebulizer pressure 45 psi, and fragmentor voltage 150 V. Methanol was of high performance liquid chromatography (HPLC) grade (Baker, Milan, Italy); hexane, ethyl acetate, dichloromethane and ethanol were of gas chromatography grade purchased from Baker (Milan, Italy); The aluminium TLC plates 20 × 20 cm silica gel 60 F254, 0.25 mm were purchased from Merck, while the silica gel grade 70-230 mesh, 60 Å was purchased from Sigma Chemical Co., Inc. Formic acid used as mobile phase additive in LC-MS analysis was obtained from Fluka/Sigma-aldrich (Italy).

### 3.2. Plant material

Ripe fruits of *M. azedarach* were collected in Thessaloniki, Greece in February 2007. A voucher specimen was deposited at the Department of Ecology, School of Biology, Aristotle Univeristy of Thessaloniki, Greece for species identification by the Prof. D. Vokou.

### 3.3. Extraction and isolation

The ripe lyophilized powdered fruits of *M. azedarach* (550 g) were extracted with *n*-hexane (16.5 L) in a Soxhlet apparatus to remove fatty acids (19.9 g). The solid residue was extracted with MeOH to give, after concentration, 200 g of dry extract. Then, the MeOH extract was suspended to methanol-water (50:50 v/v) and partitioned with DCM to afford a DCM soluble fraction. After concentration, the resulting extract (20 g) was subjected to open column chromatography (CC) (silica gel, 2,100 g) using a step gradient polarity elution. Twenty six L of mixtures of various solvents (2 L each eluent) of increasing polarity [*n-*hexane, DCM, ethyl acetate (EtOAc), and MeOH], beginning from a 100% *n-*hexane and reaching 100% MeOH, were used for the column’s gradient elution to afford sixty five fractions of 400 mL each. Fractions 1A-1E, *n-*hexane eluate; fractions 2A-2E, *n-*hexane-DCM (75:25) eluate; fractions 3A-3E, *n-*hexane-DCM (50:50) eluate; fractions 4A-4E, *n-*hexane-DCM (25:75) eluate; fractions 5A-5E, DCM eluate; fractions 6A-6E, DCM: EtOAc (75:25) eluate; fractions 7A-7E, DCM-EtOAc (50:50) eluate; fractions 8A-8E, DCM-EtOAc (25:75) eluate; fractions 9A-9E EtOAc eluate; fractions 10A-10E, EtOAc-MeOH (75:25) eluate; fractions 11A-11E, EtOAc-MeOH (50:50) eluate; fractions 12A-12E, EtAOc-MeOH (25:75); fractions 13A-13E, methanol eluate. The collected fractions were evaporated under vacuum and examined by TLC. Homogeneous fractions were pooled to give three major fractions (F1-F3). F1 (506 mg) was subjected to CC (silica gel, 50 g) with 620 mL of *n-*hexane-EtOAc (70:30 v/v) to afford 155 subfractions (F1.1-F1.155) of 4 mL each. The homogeneous subfractions F1.38-F1.43 were combined (52.25 mg) and subjected to CC (silica gel, 6 g) with 50 mL *n-*hexane-EtOAc (70:30 v/v) to afford 50 fractions of 1 mL each. The fractions 13-17 were combined and evaporated, yielding compound **4** (19.5 mg). The homogeneous subfractions F1.56-F1.63 were combined (28.07 mg) and successively purified on CC (silica gel, 6 g) with 50 mL *n-*hexane-EtOAc (70:30 v/v) to afford 50 fractions of 1 mL each. The fractions 11-13 were combined and evaporated to yield compound **3** (8.8 mg). F2 (907 mg) was rechromatographed on CC (silica gel, 90 g) with 3.7 L of *n-*hexane-EtOAc (70:30 v/v) to afford 188 subfractions (F2.1-F2.188) of 20 mL of each. The homogeneous subfractions F2.40-F2.42 were combined, evaporated and the yielding 90.09 mg were rechromatographed on CC (silica gel, 9 g) with 50 mL hexane-EtOAc (80:20 v/v) to afford 50 fractions of 1 mL each. Fractions 15-24 were combined and evaporated, yielding compound **1** (42.9 mg). F3 (975 mg) was rechromatographed on CC (silica gel, 95 g) with 3.8 L of hexane-EtOAc (70:30 v/v) to afford 188 subfractions (F3.1-F3.188) of 20 mL each. The subfractions F3.81-F3.87 were combined and evaporated, yielding 108 mg that were rechromatographed on CC (silica gel, 10 g) with 100 mL of DCM-EtOAc (90:10 v/v) to afford 100 fractions of 1 mL each. The fractions 45–75 were combined and evaporated yielding compound **2** (71.5 mg).

### 3.4. Spectroscopic data

*3-α-Tigloylmelianol* (**1**). white amorphous powder; m.p. 194–195 ºC; UV (MeOH): λ_max_ nm (log ε) = 209.9(3.45); HR-ESI MS (*m/z*) 555.4093 [M + H]^+^ (calcd. 555.4044 ); ESI MS *m/z* (rel. int.): 552 (12) [M]^+^, 537 (90) [M – H_2_O + H]^+^, 577 (84) [M + Na]^+^, 1131 (100) [2M + Na]^+^; ^1^H- and ^13^C-NMR data, see Table 2.

### 3.5. Test for in vitro cytotoxic activity

Cell viability was determined as previously reported [38]. Briefly, cell viability in the presence of the triterpenoids was determined using the cleavage of the tetrazolium salt MTT (3-(4,5-dimethylthiazol-2-yl)-2,5-diphenyltetrazolium bromide) by the mitochondrial enzyme succinate dehydrogenase to give a blue product (formazan). The absorbance of each well was measured on an Eurogenetics MPR-A 4i microplate reader using a test wavelength of 570 nm and a reference wavelength of 630 nm, by duplicate. Results were expressed as a percentage of absorbance of treated cells with respect to untreated ones. The CC_50_ was defined as the concentration of compound that caused a 50% reduction in cell viability. 

### 3.6. Test for in vitro antiproliferative activity

2.4 × 10^6^ cells were seeded in 96-well plates together with different concentrations of the triterpenoids in duplicate, and incubated at 37 ºC for 24 h in 4% CO_2_ atmosphere. Cells were then fixed with 10% formaldehyde for 15 min at room temperature, washed once with distilled water and stained with 0.05% crystal violet in 10% ethanol for 30 min. Afterwards cells were washed once, and eluted with a solution of 50% ethanol and 0.1% acetic acid in water. The absorbance of each well was measured on an Eurogenetics MPR-A 4i microplate reader using a test wavelength of 590 nm. The IC_50_ was defined as the concentration of compound that caused a 50% reduction in cell survival.

### 3.7. Second stage juveniles (J2) paralysis bioassays

Freshly hatched *M. incognita* J2 (24 h) were extracted from infested tomato roots according to Hussey and Barker (1973) [39] to be used for the experiments. Melianone, 3-*α*-tigloylmelianol, 21-*β*-acetoxymelianone and methyl kulonate, were individually subjected to dose response experiments against J2 at the dose range of 31.2–500 μg/mL. In the frame of our research testing limonoids for nematicidal activity, limonin was also subjected in a dose response experiment. Stock solutions were prepared in dimethyl sulfoxide (DMSO) and further dilutions were made in distilled water. Final concentration of DMSO never exceeded 1% v/v. The bioassays were carried out in Cellstar^® ^96-wellcell culture plates (Greiner bio-one) and each treatment was represented by 25 J2 per well. Plates were covered with plastic lids and were maintained in dark at 28 ºC. Juveniles were observed with the aid of an inverted microscope (Euromex, Holland) at 40× and were ranked into two distinct categories: motile or paralyzed. The paralysis experiments were performed twice, and every treatment was replicated per experiment six times. Assessments were made 1day (1d) and 4d after bioassay’s start.

### 3.8. Statistical analysis

Since paralysis in solvent (DMSO) was not significantly different from that observed in distilled water, the percentages of paralyzed J2 recorded in the microwell assays were corrected by eliminating the natural death/paralysis in the water control according to the Schneider Orelli’s formula (Puntener, 1981) [40]: Corrected % = {(Mortality % in treatment-Mortality % in control)/(100-Mortality % in control)}×100 and they were analyzed (ANOVA) after being combined over time. Since ANOVA indicated no significant treatment by time interaction, means were averaged over experiments. Treatment means were compared by Duncan's multiple range tests.

## 4. Conclusions

A new tirucallane triterpenoid, 3-α-tigloylmelianol (**1**), was isolated along with three known tirucallanes (**2**-**4**) [25,26,27,28,29] from the dichrolomethane-soluble part of the methanol extract obtained from the fruits of *Melia azedarach*. 21-β-acetoxy-melianone (**3**), 3-α-tigoylmelianol (**1**) and melianone (**2**) were cytotoxic while 21-β-acetoxymelianone (**3**), and 3-α-tigloylmelianol (**1**) showed an additional moderate antiproliferative effect against the human lung adenocarcinoma epithelial cell line A549, suggesting *M. azedarach* potential for further investigation as a natural source of anticancer agents. Similarly to azadirachtin [37], neither the Chinaberry limonoids nor limonin were found to paralyse *M. incognita* juveniles which fact implies that such activity [8] lies elsewhere in the methanol (MeOH) extract obtained from the ripe fruits of *M. azedarach.*
